# Enhancing cancer drug discovery: QSAR modeling with machine learning and chemical representations

**DOI:** 10.1371/journal.pone.0343654

**Published:** 2026-03-17

**Authors:** Raúl Acosta-Murillo, José Carlos Ortiz-Bayliss, Patricio Adrian Zapata-Morin

**Affiliations:** 1 Department of Microbiology and Immunology, School of Biological Sciences, Universidad Autónoma de Nuevo León, Pedro de Alba SN, San Nicolás de los Garza, Nuevo León, Mexico; 2 Tecnologico de Monterrey, School of Engineering and Sciences, Eugenio Garza Sada, Monterrey, Nuevo León, Mexico; National Library of Medicine, UNITED STATES OF AMERICA

## Abstract

Accurately predicting the bioactivity of small molecules against cancer therapeutic targets remains a significant challenge at the intersection of cheminformatics and drug discovery. This study comprehensively evaluates chemical representations, including AtomPair Counts (APC),Avalon (AVN), Extended-Connectivity Fingerprint diameter 4 (ECFP4), Extended-Connectivity Fingerprint diameter 6 (ECFP6), Feature-based Morgan 2 (FM2), Feature-based Morgan 3 (FM3), Mol2Vec (M2V), Molecular ACCess System (MACCS), Mordred 2D Chi Kappa (MK2), RDKFingerprint (RDF), Rdkit PhysChem (RDC), Torsion (TSN) combined with machine learning algorithms (Bayesian Ridge (BRG), Elastic Net (ENT), Extra Trees (ETT), Hist Gradient Boosting (HGT), K-Nearest Neighbors (*k*NN), Lasso (LSS), Multi-layer Perceptron (MLP), Partial least squares (PLS), Random Forest (RFT), Ridge (RDG), Support Vector Regressor (SVR), and XGBoost (XGB)) for predicting cancer bioactivities. The results show that while AVN chemical representation, in conjunction with SVR algorithm, achieved the highest predictive accuracy, with *R*^2^ of 0.735 in FGFR1 dataset; The mTOR dataset demonstrated the highest average performance across all models and chemical representations, with an *R*^2^ of 0.592 across various cancer datasets. These findings demonstrate how cheminformatics tools like molecular fingerprints and quantitative structure-activity relationship (QSAR) modeling can significantly enhance bioactivity prediction, ultimately contributing to more efficient and targeted cancer drug discovery.

## Introduction

### Overview of QSAR modeling and machine learning in drug discovery

QSAR models have been crucial in drug discovery, offering predictive insights into the biological activity and properties of untested compounds by predicting activity based on molecular structure [1]. These models rely on various computational approaches to establish chemical structure and biological response relationships. However, selecting appropriate descriptors and modeling techniques remains a persistent challenge, with continuous innovation and debate regarding optimizing these models for improved accuracy and utility in drug discovery [2]. In recent years, machine learning (ML) techniques have increasingly been integrated with QSAR modeling to enhance predictive capabilities. This study evaluates the impact of these ML techniques and various molecular descriptors to identify combinations that optimize the predictive accuracy of QSAR models [3–5].

### Challenges in predicting bioactivity for cancer therapeutics

Cancer remains one of the most significant global health challenges, with breast, prostate, and lung cancers among the most prevalent and deadly [6,7]. Accurate bioactivity prediction for small molecules targeting key cancer therapeutic proteins is essential for advancing cancer drug discovery. Recent research emphasizes the need to explore new therapeutic targets, such as HER2, PARP, PI3K, and mTOR, which play critical roles in cancer cell growth, proliferation, and metastasis [8]. Identifying molecules that effectively inhibit these targets requires sophisticated QSAR models and ML techniques capable of navigating the complexities of molecular structures and large datasets.

### Molecular representations and descriptors in QSAR

Representing molecules in computational models poses several challenges, especially when dealing with non-canonical or non-unique molecular representations. To address this, a diverse array of molecular encoding schemes was employed. Substructure-based keys, including MACCS keys [9] and PubChem fingerprints, were used to offer binary resolutions of structural features. These were complemented by topological descriptors such as APC [10], TSN [11], and AVN [12], which describe substructural connectivity. Higher fidelity descriptors, specifically Extended Connectivity Fingerprints (ECFP4, ECFP6) and Feature-based Morgan fingerprints (FM2, FM3) [13], were applied to capture atomic environments within defined radii. These circular fingerprints are widely used in structure-activity modeling [14–17].

To capture semantic and contextual information providing new perspectives beyond traditional fingerprints, advances in continuous vector embeddings were leveraged. These included M2V, trained via the Word2Vec algorithm [18], and SMILESVec, which capture substructural relationships [19]. Furthermore, physicochemical descriptors play a crucial role in predicting bioactivity by offering insights into ADME (Absorption, Distribution, Metabolism, and Excretion) properties [20]. To this end, the RDKit descriptor suite and Mordred calculator were used to generate constitutional, electronic, and topological indices (e.g., Chi, Kappa, E-State) [21].

### Dimensionality reduction techniques

Given the complexity and high dimensionality of molecular descriptors, effective feature selection is essential to simplify data analysis and enhance model robustness. Instead of dimensionality reduction techniques like Principal Component Analysis (PCA), a stepwise feature filtering approach was employed to eliminate redundancy while preserving the original physicochemical meaning of the descriptors. First, invariant features were removed using a zero-variance filter (VarianceThreshold). Subsequently, a custom correlation filter was applied to identify and remove highly collinear features (Pearson correlation coefficient >0.95 ) [22,23]. Importantly, to prevent data leakage, this feature selection process was fitted only on the training dataset prior to any cross-validation. The specific list of retained features identified in the training phase was then consistently applied to all held-out folds and external test sets. This strict separation ensures that the models are not biased by information from the test data, addressing common pitfalls in QSAR modeling such as overfitting and inflated performance estimates [24,25].

### Machine learning models in QSAR

The integration of ML algorithms has fundamentally transformed QSAR modeling. While traditional linear approaches such as Ridge and Lasso regression established the foundation for interpreting physicochemical relationships [26,27], modern non-linear methods have significantly expanded predictive capabilities. Tree-based ensemble method, most notably Random Forest (RFT) and Gradient Boosting frameworks like XGBoost have emerged as some of the most effective tools, offering robust accuracy and computational efficiency in predicting bioactive properties [5,28,29]. Concurrently, kernel-based methods such as SVR and probabilistic approaches like Gaussian Processes have provided powerful alternatives for modeling complex non-linear landscapes [30,31]. More recently, deep neural networks (DNNs) have been applied to further push the boundaries of prediction, although their incremental improvements in accuracy compared to established ensemble models often come at the cost of increased computational complexity and reduced interpretability [32].

Recently, graph neural networks (e.g., GCNs, GATs, MPNNs, AttentiveFP) have enabled end-to-end molecular graph learning that often matches or surpasses RFT/XGB performance on large, multi-task QSAR datasets, albeit at higher GPU-dependent training costs [33,34]. Concurrently, transformer-based models such as ChemBERTa, the Molecule Attention Transformer, and MolE leverage self-attention on Simplified Molecular Input Line Entry System (SMILES) or graph embeddings to deliver state-of-the-art QSAR predictions after pre-training on millions of unlabeled molecules, though they demand substantial computational and memory resources [35,36]. Finally, interpretability methods like SHAP and attention-weight visualization decompose activity predictions into atom- and fragment-level contributions, bolstering medicinal chemists‘ confidence and guiding rational lead optimization [35,37].

### Objectives and research questions

This work comprehensively evaluates machine learning models and molecular representations for predicting the bioactivity (pIC50) of small molecules targeting various cancer therapeutic proteins. We employ multiple sets of molecular descriptors to compare their performance in QSAR modeling. The main research question is: *Which combination of molecular representations and machine learning algorithms provides the highest predictive accuracy for bioactivity prediction of small molecules against cancer targets?*

## Materials and methods

This section details the datasets, chemical representations, feature engineering techniques, machine learning models, and validation methods used in this study.

### Data acquisition and preprocessing

Bioactivity data for sixteen therapeutic targets were obtained from the ChEMBL database [38], specifically focusing on IC_50_ measurements, which quantify the half-maximal inhibitory concentration of a compound. To ensure data consistency, we excluded records containing inequality relations (>, < ), as well as records with missing values. This filtering step was crucial to avoid introducing noise or uncertainty into the dataset. The molecular structures were standardized to their neutral, canonical SMILES representations using RDKit‘s Salt Remover utility, ensuring the removal of salts and other unimportant molecular components [39,40]. For compounds with multiple IC_50_ measurements, we calculated the median to represent a single, reliable activity value. We applied a ceiling of $10^{8}\;$ nM to avoid distortion from exceedingly high IC_50_ values [41]. These IC_50_ values were subsequently converted to pIC_50_ values using the formula:


$$\text{pIC}50=-\log10({\text{IC}}_{50}\times 10^{-9})\;$$ (1)

### Chemical representation

The core of the QSAR modeling process lies in the choice of molecular representations. In this study, we applied multiple molecular encoding schemes to capture diverse structural and physicochemical features of the compounds. These included molecular fingerprints such as ECFP4, ECFP6, FM2, FM3, and RDKIT Fingerprints [13], as well as topological fingerprints like MACCS [9], APC [10], TSN [11], and AVN [42], which describe substructural and connectivity information. Additionally, physicochemical descriptors derived from RDKit‘s built-in 2D descriptor suite were computed [40], alongside Mordred-calculated descriptors such as MK2, which describe molecular constitutional, electronic, and topological features [21].

To add more diversity to the molecular representations, we also used Mol2Vec embeddings. These are vector-based representations of molecules created using a Word2Vec model, which helps capture relationships between molecular substructures [18].

### Feature engineering

Given the high dimensionality of the chemical representations, the datasets underwent feature engineering to eliminate irrelevant features. We applied the zero-variance filter, removing features with zero variance using VarianceThreshold [25]. Later, we used a custom-built correlation filter to remove highly correlated features, which can introduce multicollinearity and negatively impact model performance. The filter kept only the first feature in each highly correlated pair (with a Pearson correlation coefficient greater than 0.95), discarding the others. This step helped reduce redundancy and improve model interpretability [23]. Finally, we standardized the features using *Z*-score normalization (except for tree-based models, which are invariant to scaling), ensuring that all features had a comparable scale for models sensitive to feature magnitude, such as linear regression and support vector regressor (SVRs) [43].

### Machine learning algorithms

In this study, we chose traditional machine learning methods for their interpretability and relatively low computational demands, aligning with our goal of establishing robust baseline models for molecular bioactivity prediction. Our methodology involved using various machine learning algorithms, including traditional models such as *k*NN [44] and PLS [45], alongside advanced techniques like SVR [46] RFT [47], and XGB [48]. Model training was conducted with the Scikit-Learn [25] and XGBoost [29] libraries.

While deep learning (DL) has shown potential in QSAR applications, recent studies, such as the IDG-DREAM Drug-Kinase Binding Challenge (2019), suggest that DL’s predictive improvement over traditional methods like XGB or RFT can be modest [5]. This limited performance advantage, combined with the high computational demands and interpretability challenges associated with DL, led us to focus on traditional approaches in this phase of our study.

*k***-nearest neighbors (***k***NN):** A non-parametric method that predicts outcomes based on proximity to the *k* closest points in the training data [49]. The number of neighbors was optimized over the set k∈{3,5,7,11,15} .

**Partial least squares (PLS):** A technique that handles multicollinearity by extracting latent factors that maximize the covariance between independent and dependent variables [50]. The model was tuned by selecting the optimal number of latent components from {2,5,10} .

**Support vector regressor (SVRs):** SVRs create a hyperplane that maximizes the margin or minimizes regression errors using kernel functions [51].We utilized the Radial Basis Function (RBF) kernel, optimizing the regularization parameter *C* (log-scale $10^{-2}\;$ to $10^{3}\;$) and the kernel coefficient *γ* (log-scale $10^{-4}\;$ to $10^{0}\;$).

**Random Forest (RFT):** RFT creates an ensemble of decision trees with random feature selection [52]. Hyperparameters tuned included the number of estimators ({100,300} ) and the maximum features considered for splitting ($\sqrt{n\_features}\;$ or $\log_{2}n\_features\;$).

**Extreme Gradient Boosting (XGB):** XGB sequentially builds weak learners to reduce residual errors [29]. The grid search included the number of estimators ({200,500} ), learning rate ({0.01,0.05,0.1} ), and maximum tree depth ({3,6,9} ).

**ElasticNet (ENT):** A linear model combining L1 and L2 regularization [53]. Optimization was performed on the regularization strength *α* ($10^{-4}\;$ to $10^{2}\;$) and the L1 ratio ({0.1,0.5,0.9} ) to balance Lasso and Ridge penalties.

**ExtraTrees (ETT):** An ensemble method using extremely randomized trees [54]. Similar to RF, we optimized the number of estimators ({100,300} ) and the feature subset size for splitting ($\sqrt{n\_features}\;$ or $\log_{2}n\_features\;$).

**HistGBDT (HGT):** A histogram-based Gradient Boosting method that bins continuous features for speed [55]. We tuned the maximum number of iterations ({200,500} ) and the learning rate ({0.01,0.05,0.1,0.2} ).

**Lasso (LSS):** A regression analysis method that performs variable selection using L1 penalties [27]. The regularization strength *α* was optimized on a logarithmic scale.

**Multi-layer Perceptron (MLP):** A feedforward artificial neural network trained via backpropagation [56]. The architecture search included single hidden layers of 50 or 100 neurons and a two-layer configuration (100,50, with L2 regularization $\alpha \in \{10^{-4},10^{-2}\}\;$.

**Ridge (RDG):** A linear regression model with L2 regularization [26]. The regularization strength *α* was optimized over a logarithmic scale ranging from $10^{-4}\;$ to $10^{4}\;$.

**Bayesian Ridge (BRG):** A probabilistic linear model that estimates coefficients with Gaussian priors [57]. This model self-tunes regularization parameters during fitting without an external grid search.

### Training and hyperparameter optimization

The pipeline supports 12 distinct machine learning models, spanning a variety of algorithmic paradigms. These include linear models such as Ridge, Lasso [27], ENT, and BRG; tree-based ensemble methods such as RFT [28], ETT, HGT, XGB [29], SVR [30]; and other varied methods such as *k*-NN [58], and multi-layer perceptron neural networks [56]. We conducted hyperparameter optimization for each model using Halving Randomized Search Cross-Validation, which efficiently narrows the search space by iteratively halving the number of candidate configurations based on performance [59]. We employed a group three-fold cross-validation scheme with Murcko scaffolds [60] as grouping variables, to ensure that training and validation sets did not overlap in scaffold composition, preventing data leakage and reducing the risk of overfitting [41]. We tested 20 candidate hyperparameter sets per model, and identified the best-performing configurations. Upon finding optimal configurations, we retrained each model on the entire training set. We evaluated them on an unseen test set comprising approximately 15% of the molecules to ensure unbiased performance assessment [61–63].

### Model evaluation and statistical analysis

To evaluate model performance, we used the coefficient of determination (*R*^2^) as the primary metric, computed on an external test set generated using Murcko scaffold splitting to ensure that test compounds possessed distinct molecular frameworks from the training set [60]. Outliers were identified and removed from the predicted registry using the 1.35 Interquartile Range (IQR) technique, ensuring that extreme predictions did not distort the results [64]. In addition to *R*^2^, we calculated RMSE and Mean Absolute Error (MAE) to provide a comprehensive view of prediction accuracy and error magnitude. The distribution of *R*^2^ scores was analyzed using the Friedman test to identify global performance differences between models and representations [65]. We conducted this using the scipy.stats.friedmanchisquare function [66]. Where significant differences were identified (*p*-value <  0.05), we conducted the Nemenyi post-hoc test to assess pairwise differences, visualizing the resulting *p*-values via heatmaps. Additionally, pairwise T-tests were conducted to determine statistical significance between specific model and representation pairs. To investigate model stability and consistency, we performed a ranking analysis based on median *R*^2^ scores across datasets. We complemented this by analyzing the trade-off between performance and variability, computing the standard deviation of *R*^2^ (model disagreement) and determining dataset difficulty, defined as $(1-\text{Best }R^{2}$ based on Cohen’s framework [67]. These metrics allowed us to distinguish between stable predictive power and high variability across the different cancer therapeutic targets.

## Results

**Fig 1:**
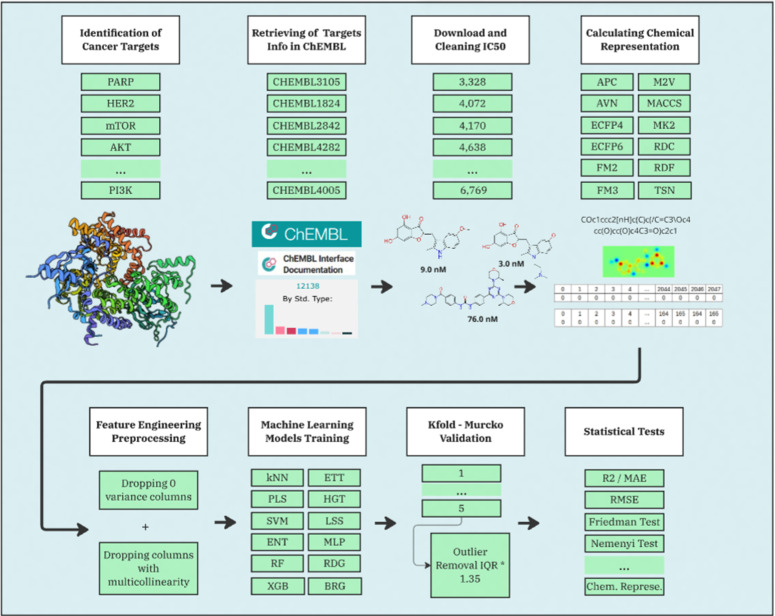
Workflow diagram of the QSAR modeling pipeline incorporating chemical representations and machine learning algorithms. Methodological process diagram detailing the research workflow, including dataset selection, chemical representations (such as APC, AVN, ECFP4, ECFP6, FM2, FM3, M2V, MACCS, MK2, RDF, RDC, TSN), and machine learning algorithms applied (BRG, ENT, ETT, HGT, *k*NN, LSS, MLP, PLS, RFT, RDG, SVR, XGB). The results were validated, ensuring robust model performance evaluation across all tested combinations.

To evaluate the influence of molecular representations, machine learning algorithms, and preprocessing strategies, we selected 16 biological targets associated with breast, prostate, and lung cancers [8,68]. The bioactivity data for these targets were curated and encoded using 12 types of molecular representations [16,19]. Data preprocessing included removing low-variance features and eliminating multicollinearity. Furthermore, supervised feature selection was applied using univariate statistical tests and Lasso-based importance. We used these processed features to train 12 machine-learning QSAR models [5], with hyperparameters optimized via a randomized search strategy. This workflow resulted in a comprehensive set of trained models, as illustrated in Fig 1. Finally, we conducted statistical tests to assess significant differences in predictive performance.

After scouring the ChEMBL database, we identified 16 distinct cancer biological targets, each accompanied by its ChEMBL ID, UniProt ID, gene, gene description, and the cancer they are related to, as presented in Table 1. Additionally, we provide the number of IC50 entries for each target. The ChEMBL datasets vary in size, ranging from 2,444 entries for the CHK1 protein to 16,715 entries for the EGFR1 receptor. On average, the datasets contain 5,625 entries.

**Table 1:** Description of the identified cancer targets with ChEMBL and UniProt IDs.

ChEMBL ID	Gene	UniProt ID	Description	# IC50	Related
CHEMBL3105	PARP	P09874	Poly [ADP-ribose] polymerase-1	3,328	B, L, T
CHEMBL1824	HER2	P04626	Receptor protein-tyrosine kinase	4,072	B, L
CHEMBL4005	PI3K	P42336	PI3-kinase p110-alpha subunit	6,769	B, L, T
CHEMBL3130	PI3K	O00329	PI3-kinase p110-delta subunit	4,638	B, L, T
CHEMBL4282	AKT	P31749	Serine/threonine-protein kinase AKT	4,170	B, L, T
CHEMBL2842	mTOR	P42345	Serine/threonine-protein kinase	5,112	B, L, T
CHEMBL3650	FGFR1	P11362	Fibroblast growth factor receptor 1	4,820	B, L
CHEMBL2742	FGFR3	P22607	Fibroblast growth factor receptor 3	3,375	B, L
CHEMBL1871	AR	P10275	Androgen Receptor	3,670	B, L
CHEMBL203	EGFR1	P00533	Epidermal growth factor receptor	16,715	B, L
CHEMBL1957	IGFR1	P08069	Insulin-like growth factor I receptor	4,424	B, L, T
CHEMBL4630	CHK1	O14757	Serine/threonine-protein kinase Chk1	2,444	B, L, T
CHEMBL279	VEGFR2	P35968	Vascular endothelial growth factor receptor 2	14,087	B, L, T
CHEMBL4722	AURKA	O14965	Serine/threonine-protein kinase Aurora-A	3,725	B, L, T
CHEMBL2185	AURKB	Q96GD4	Serine/threonine-protein kinase Aurora-B	2,367	B, L, T
CHEMBL1865	HDAC6	Q9UBN7	Histone deacetylase 6	6,287	B, L, T

### Impact of the chemical representation on QSAR performance

**Fig 2:**
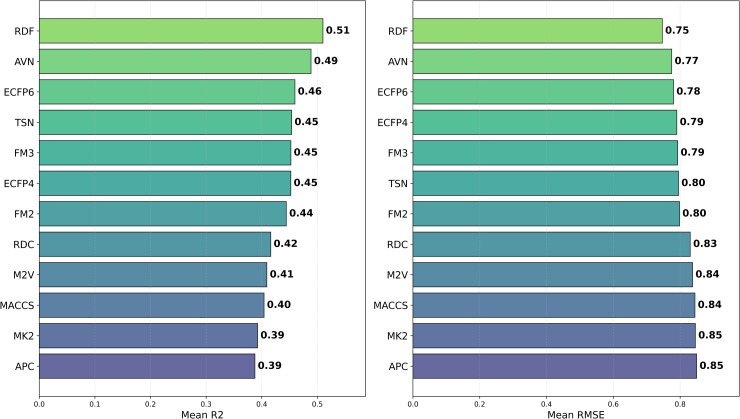
Distribution of $R^{2}\;$ and RMSE for pIC50 Across Chemical Representations. Barplot (left) showing average *R*^2^ and (right) MAE of prediction values for pIC50 across multiple cancer therapeutic datasets and model architectures, organized by chemical representations.

**Fig 3:**
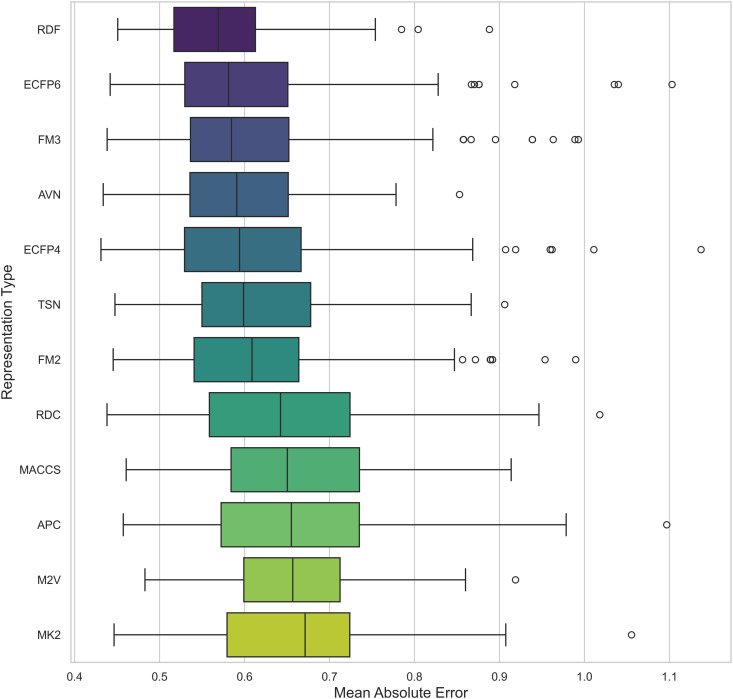
Distribution of MAE for pIC50 Across Chemical Representations. Boxplot showing the average MAE of prediction values for pIC50 across multiple cancer therapeutic datasets, organized by chemical representations.

**Fig 4:**
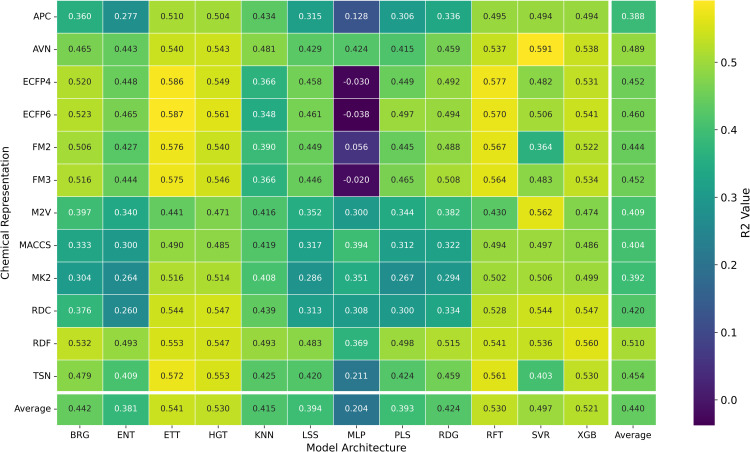
Distribution of *R*^2^ Prediction Values for pIC50 Across Chemical Representations and Model Architectures. Heatmap showing the average *R*^2^ prediction values for pIC50 across multiple cancer therapeutic datasets, organized by chemical representations and model architectures.

**Fig 5:**
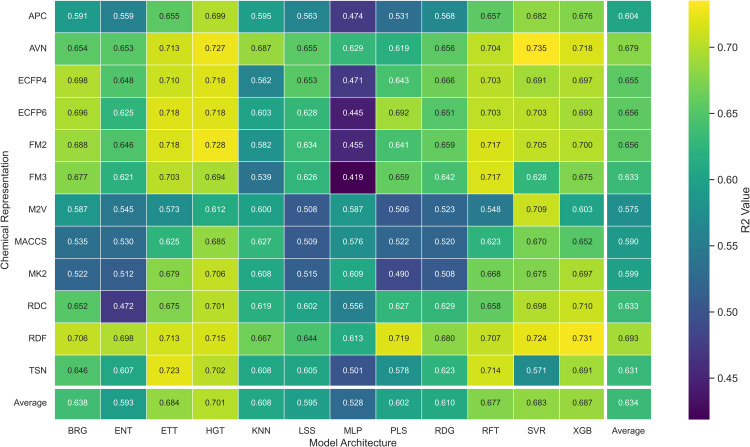
Distribution of *R*^2^ Prediction Values for pIC50 Across Chemical Representations and Model Architectures. Heatmap showing the maximum *R*^2^ prediction values for pIC50 across multiple cancer therapeutic datasets, organized by chemical representations and model architectures.

To investigate how different chemical representations impact QSAR model performance, we evaluated a range of molecular descriptors. These included various molecular fingerprints, such as ECFP4, ECFP6, FM2, FM3, and RDKit Fingerprints [13], as well as topological fingerprints like MACCS [9], APC [10], TSN [11], and AVN [42]. We also assessed RDKit descriptors [40] and Mordred-calculated descriptors [21]. The average *R*^2^ values for each chemical representation across all datasets and models are displayed in Table 2 and Fig 2, while Fig 3 presents the distribution of the MAE. Additionally, Fig 4 presents a heatmap of *R*^2^ values, providing an overview of average performance across both representation languages and model architectures (averaged across datasets). While In the Fig 5 it can be observed the best *R*^2^ fount across the datasets.

**Table 2:** Comparison of *R*^2^ and RMSE Performance for pIC50 Across Different Chemical Representations. Values represent the coefficient of determination (*R*^2^) and RMSE. The highest-performing representation (highest *R*^2^ and lowest RMSE) is indicated in bold.

Representation	*R* ^2^	RMSE
APC	0.3877	0.8495
AVN	0.4887	0.7749
ECFP4	0.4522	0.7902
ECFP6	0.4596	0.7802
FM2	0.4441	0.7981
FM3	0.4523	0.7927
M2V	0.4092	0.8373
MACCS	0.4041	0.8444
MK2	0.3925	0.8456
RDC	0.4162	0.8303
RDF	**0.5101**	**0.7466**
TSN	0.4538	0.7951

**Fig 6:**
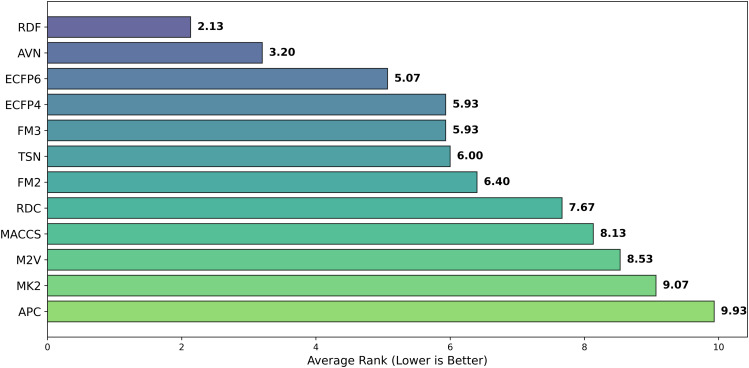
Average Ranking of *R*^2^ for pIC50 Across Chemical Representations. Barplot showing the average ranking based on *R*^2^ for pIC50 across multiple cancer therapeutic datasets and model architectures, organized by chemical representations.

**Fig 7:**
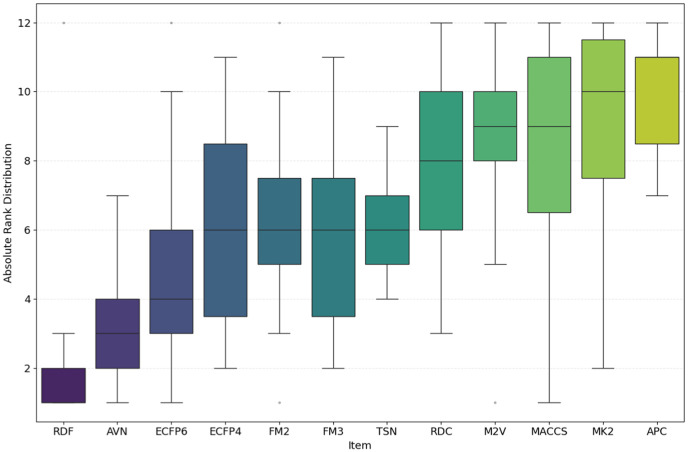
Ranking Distribution of *R*^2^ for pIC50 Across Chemical Representations. Boxplot showing the ranking based on *R*^2^ for pIC50 across multiple cancer therapeutic datasets and model architectures, organized by chemical representations.

**Fig 8:**
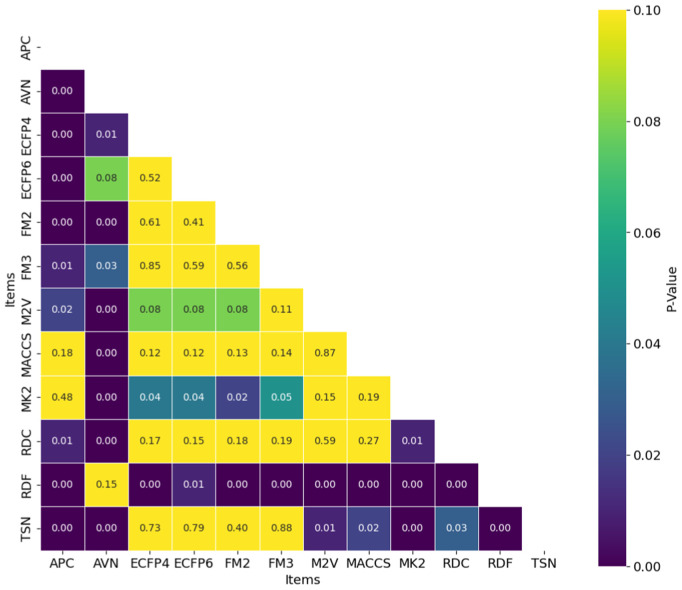
Distribution of T-test *p*-values for Statistical Significance of $R^{2}\;$ Across Chemical Representations and Model Architectures. Heatmap showing T-test *p*-values for statistical significance of *R*^2^ across multiple cancer therapeutic datasets, organized by chemical representations.

**Fig 9:**
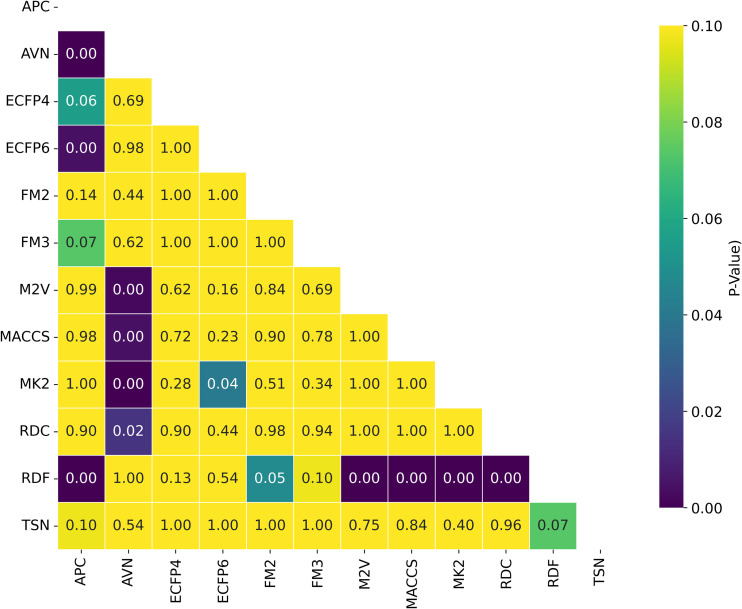
Distribution of Nemenyi p-values for Ranking Statistical Significance Across Chemical Representations and Model Architectures. Heatmap showing Nemenyi *p*-values for ranking statistical significance across multiple cancer therapeutic datasets, organized by chemical representations.

While the best *R*^2^ was observed in combination of AVN and SVR (0.591); In average, the RDF molecular fingerprint emerged as the top performer, achieving the highest average *R*^2^ value of 0.510 across datasets, and model architectures. It was closely followed by AVN (0.489) and ECFP6 (0.460). This ranking is also illustrated in Fig 6 and supported by the stability analysis in Fig 7, where the average ranks for the representations are as follows: RDF (2.13), AVN (3.20), and ECFP6 (5.07). This suggests that RDF was the most effective representation for capturing the relationship between chemical structures and biological activity in our study. Statistically significant differences were observed, with the exception of the comparison between RDF (1^st^) and AVN (2^nd^), as detailed in Figs 8 and 9.

### Impact of the machine learning model on QSAR performance

**Fig 10:**
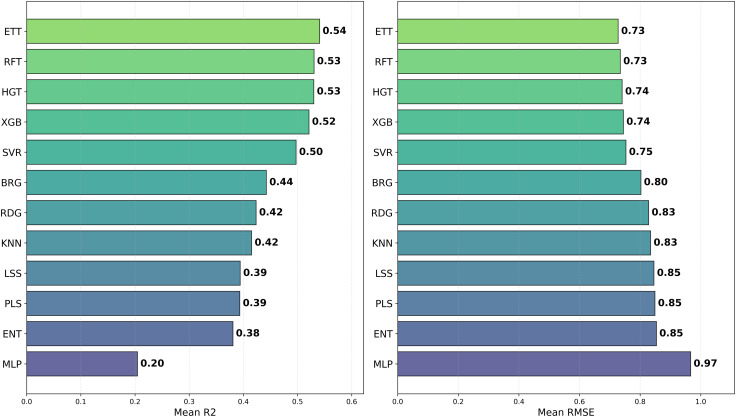
Distribution of $R^{2}\;$ and RMSE for pIC50 Across Model Architectures. Boxplot (left) showing average *R*^2^ and (right) MAE of prediction values for pIC50 across multiple cancer therapeutic datasets and chemical representations, organized by model architecture.

**Fig 11:**
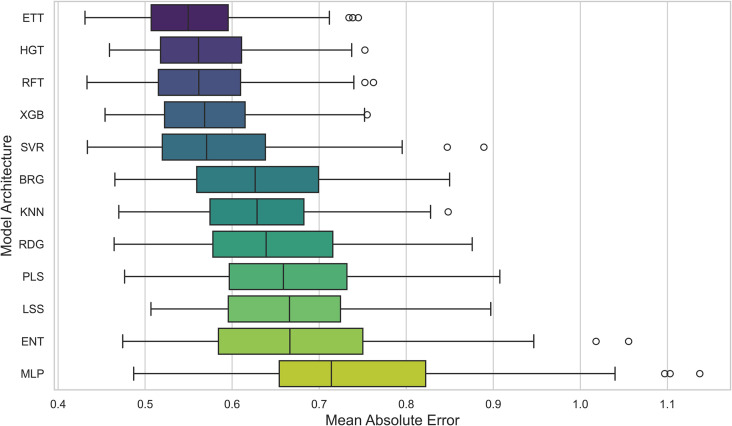
Distribution of MAE for pIC50 Across Model Architectures. Boxplot showing the average MAE of prediction values for pIC50 across multiple cancer therapeutic datasets, organized by model architecture.

To further explore the role of machine learning models in QSAR performance, we tested a suite of 12 algorithms trained for pIC50 prediction: BRG, ENT, ETT, HGT, *k*NN, LSS, MLP, PLS, RFT, RDG, SVR, and XGB (see Methods for additional details). The average *R*^2^ and RMSE values for each model across all datasets and chemical representations are provided in Figs 3 and 10, while we show the distribution of MAE values in Fig 11. As in the previous analysis, Fig 4 visualizes the heatmap of *R*^2^ values, again averaged across the datasets. In the Fig 5 it is shown the best *R*^2^ fount across the datasets.

**Fig 12:**
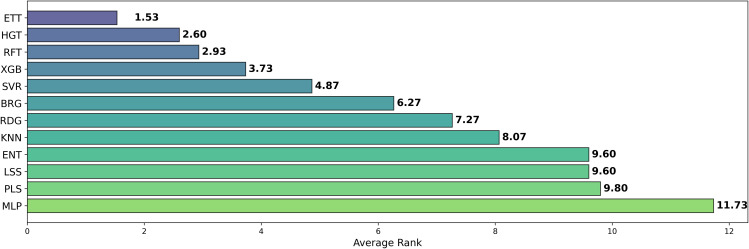
Average Ranking of $R^{2}\;$ for pIC50 Across Model Architectures. Boxplot showing the average ranking based on *R*^2^ for pIC50 across multiple cancer therapeutic datasets and chemical representations, organized by model architecture.

**Fig 13:**
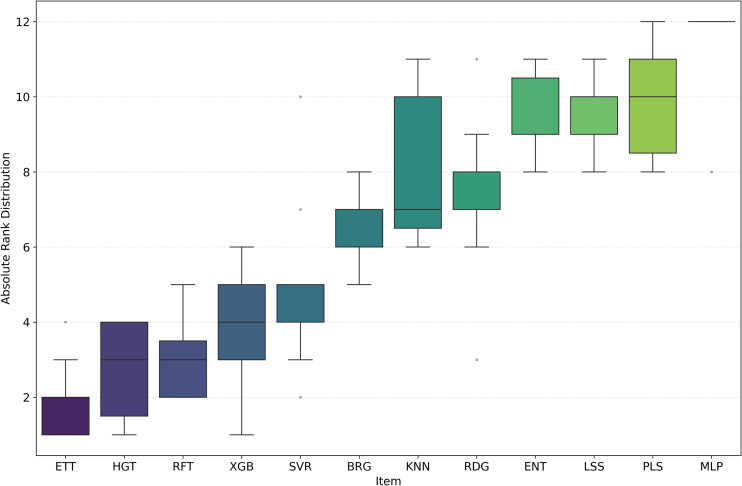
Ranking Distribution of $R^{2}\;$ for pIC50 Across Model Architectures. Boxplot showing the ranking based on *R*^2^ for pIC50 across multiple cancer therapeutic datasets and chemical representations, organized by model architecture.

**Fig 14:**
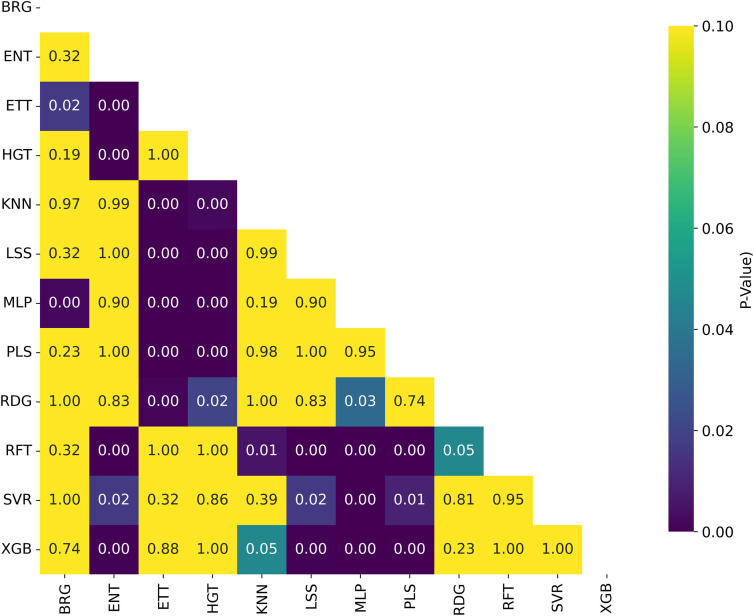
Distribution of Nemenyi p-values for Ranking Statistical Significance Across Model Architectures. Heatmap showing Nemenyi p-values for ranking statistical significance across multiple cancer therapeutic datasets and chemical representations, organized by model architecture.

**Fig 15:**
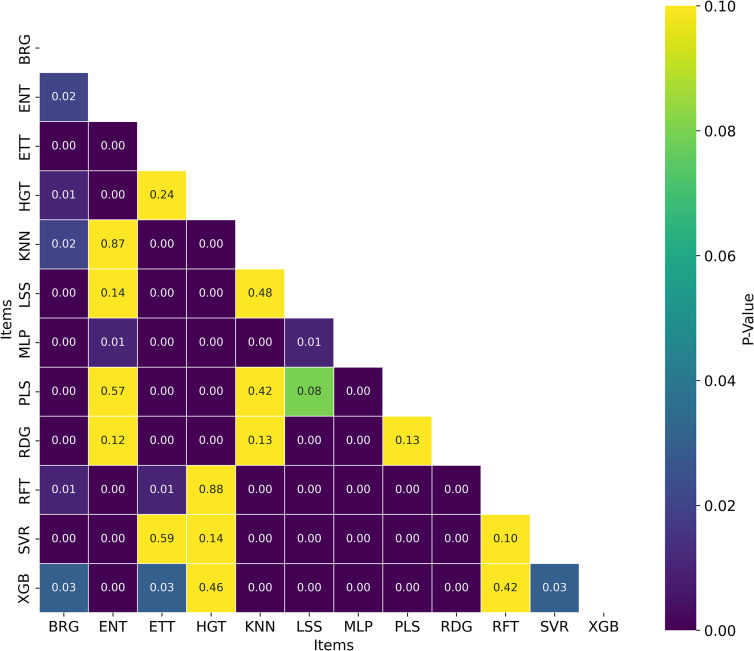
Distribution of T-test p-values for Statistical Significance of *R*^2^ Across Model Architectures. Heatmap showing T-test p-values for statistical significance of *R*^2^ across multiple cancer therapeutic datasets and chemical representations, organized by model architecture.

Among the models tested, ETT outperformed all others with the highest average *R*^2^ of 0.541, followed by HGT and RFT, both with an *R*^2^ of 0.530. This ranking is further corroborated in Fig 12 and validated by the stability analysis in Fig 13, where the average ranks for the models are: ETT (1.53), HGT (2.60), and RFT (2.2.93). Nemenyi plot between the models can be obseved at Fig 14 Statistically significant differences were found, except between ETT (1^st^), HGT and SVR, as shown in Fig 15.

### Impact of datasets on QSAR performance

**Fig 16:**
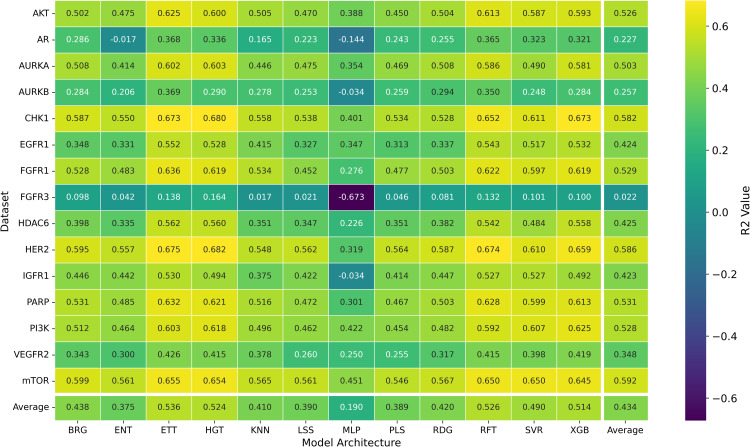
Distribution of *R*^2^ Prediction Values for pIC50 Across Datasets and Model Architectures. Heatmap showing the average *R*^2^ prediction values for pIC50 across multiple cancer therapeutic datasets, organized by chemical representations and model architectures.

**Fig 17:**
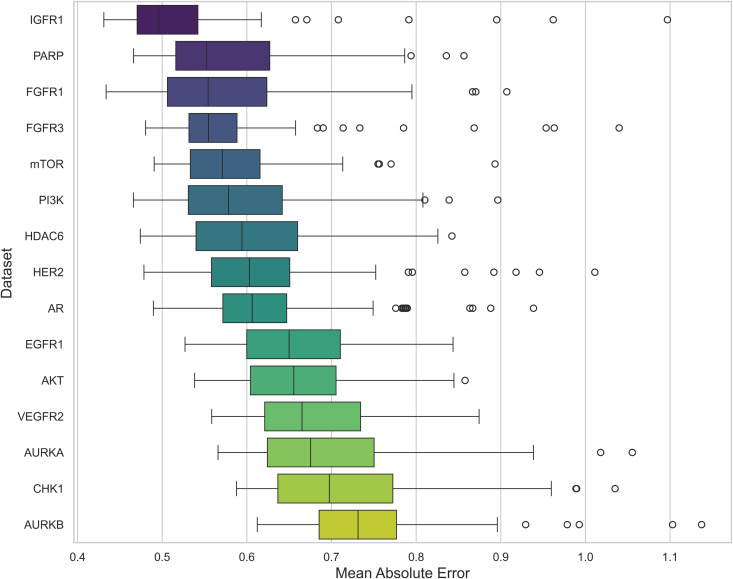
Distribution of MAE for pIC50 Across Datasets. Boxplot showing the average MAE of prediction values for pIC50 across multiple cancer therapeutic datasets, organized by dataset.

**Fig 18:**
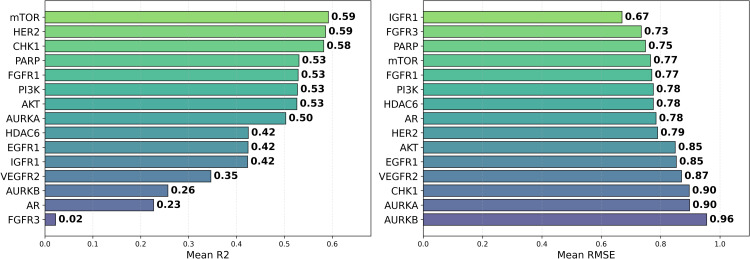
Distribution of *R*^2^ and RMSE for pIC50 Across Datasets. Boxplot (left) showing average *R*^2^ and (right) MAE of prediction values for pIC50 across multiple cancer therapeutic datasets, organized by dataset.

In addition to model performance, we also evaluated 16 different datasets for pIC50 prediction. Fig 16 again features a heatmap of *R*^2^ values, averaged across models and representations, while Fig 17 shows the distribution of MAE. The Table 4 and Fig 18 present the average *R*^2^ and RMSE values across datasets, models, and chemical representations.

**Fig 19:**
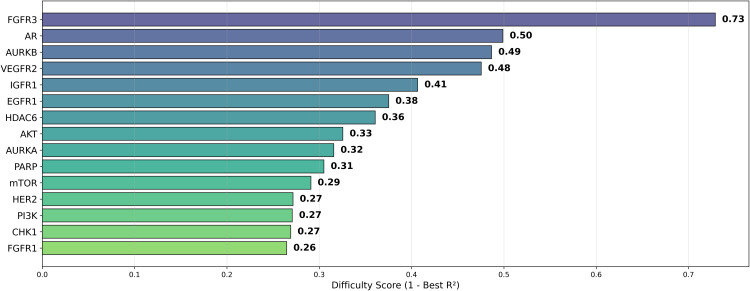
Difficulty Score (1 – *R*^2^) for pIC50 Across Datasets. Boxplot showing the difficulty score (1 – *R*^2^) for pIC50 across model architectures and chemical representations, organized by cancer therapeutic datasets.

**Fig 20:**
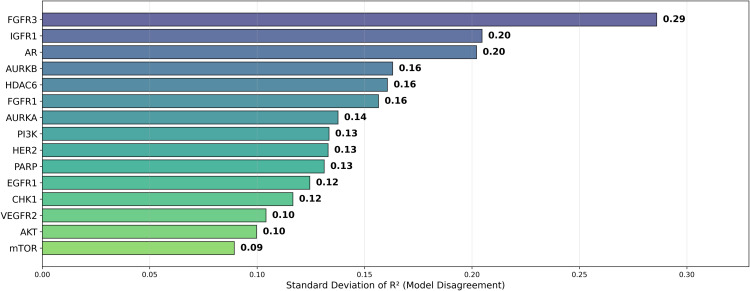
Dataset Disagreement (Standard Deviation) of Average *R*^2^ for pIC50 Across Datasets. Boxplot showing model disagreement (standard deviation) of average *R*^2^ for pIC50 across model architectures and chemical representations, organized by cancer therapeutic datasets.

**Fig 21:**
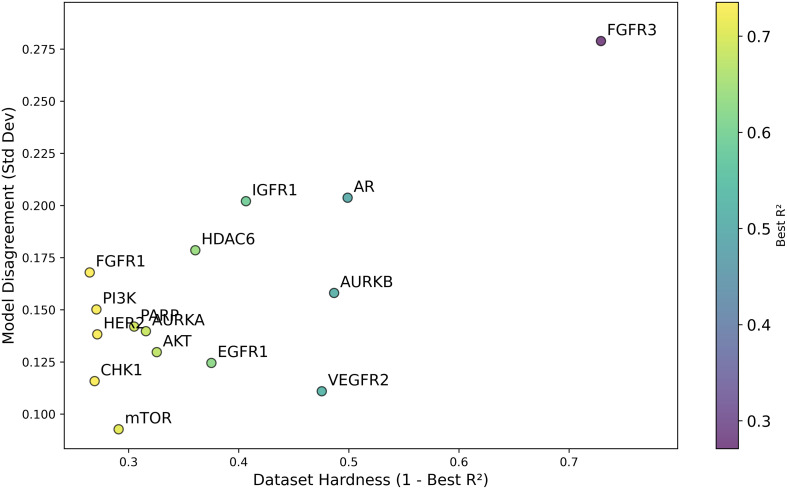
Dataset Disagreement (Standard Deviation) of Average *R*^2^ for pIC50 Across Datasets. Scatterplot showing the relationship between model difficulty (1 – best *R*^2^) and model disagreement (standard deviation) of average *R*^2^ for pIC50 across model architectures and chemical representations, organized by cancer therapeutic datasets.

Building upon Cohen’s framework [67], we define the *difficulty* of a dataset as (1 – Best *R*^2^). The difficulty scores for the various datasets are presented in Fig 19. In addition, Fig 20 illustrates the standard deviation of *R*^2^, revealing that the model with the highest average *R*^2^ did not necessarily exhibit the smallest model disagreement. This observation suggests that a higher *R*^2^ does not always imply reduced variability in model predictions, underscoring the complexity of model performance. This is further emphasized in Fig 21, where datasets with lower difficulty (on the left side) show model disagreements in the range of 0.1 to 0.2, highlighting how even less challenging datasets can exhibit considerable model variability.

**Fig 22:**
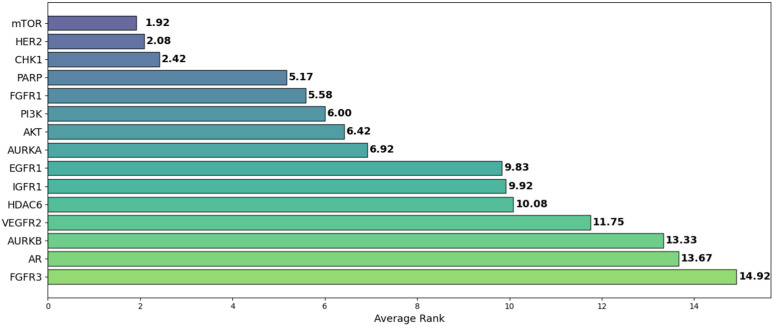
Average Ranking of *R*^2^ for pIC50 Across Datasets. Boxplot showing the average ranking based on *R*^2^ for pIC50 across model architectures and chemical representations, organized by cancer therapeutic datasets.

**Fig 23:**
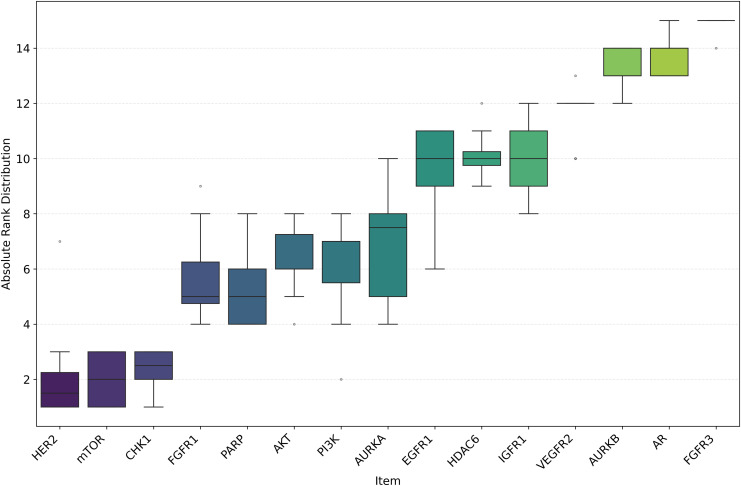
Ranking Distribution of *R*^2^ for pIC50 Across Datasets. Boxplot showing the ranking based on *R*^2^ for pIC50 across model architectures and chemical representations, organized by cancer therapeutic datasets.

**Fig 24:**
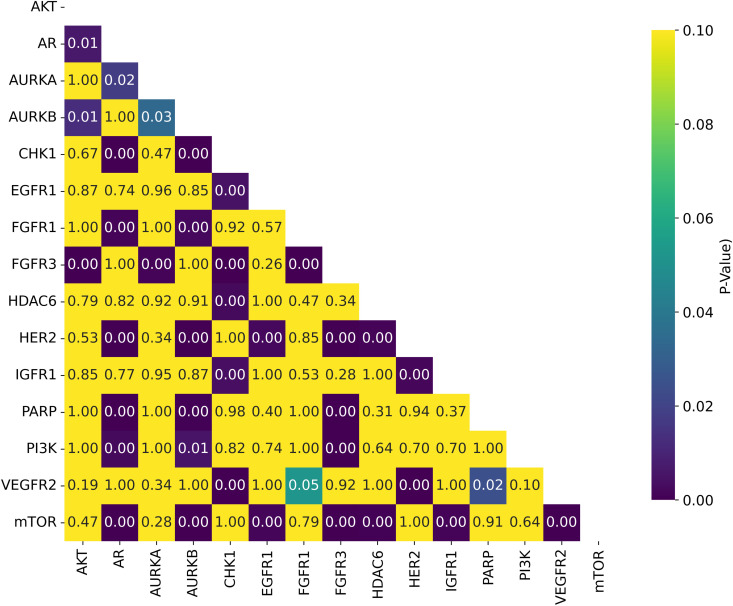
Distribution of Nemenyi p-values for Ranking Statistical Significance Across Datasets. Heatmap showing the Nemenyi p-values for ranking statistical significance across multiple cancer therapeutic datasets, organized by chemical representations and model architectures.

**Fig 25:**
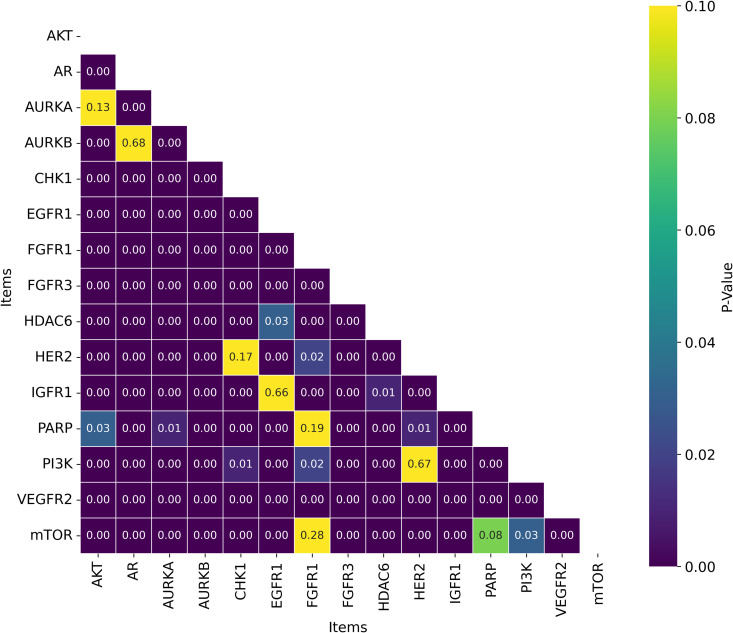
Distribution of T-test p-values for Statistical Significance of *R*^2^ Across Datasets. Heatmap showing the T-test p-values for *R*^2^ statistical significance across multiple cancer therapeutic datasets, organized by chemical representations and model architectures.

Among the datasets, mTOR demonstrated the highest average *R*^2^ of 0.592, followed by HER2 (0.586) and CHK1 (0.582), as seen in Fig 18. These results suggest that mTOR was the dataset most strongly correlated with biological activity, with a higher *R*^2^ indicating more reliable predictive power based on molecular structure [24]. This is also supported by the ranking in Fig 22 and the stability analysis in Fig 23, where the average ranks for the datasets are: mTOR (1.92), HER2 (2.08), CHK1 (2.42). Nemenyi values between datasets can be observed in Fig 24. Statistically significant differences were observed, except between mTOR (1^st^), PARP, and PI3K, as shown in Fig 25.

## Discussion

We have analyzed the impact of various chemical representations, including MACCS keys [9], PubChem fingerprints, APC [10], TSN [11], AVN [12], ECFP4, ECFP6, Feature-based Morgan fingerprints (FM2, FM3) [13], M2V [18], RDKit descriptor suite and Mordred calculator [21]. These chemical representations are characterized by their widespread adoption and efficiency. According to our experiments, RDF obtained the highest average *R*^2^ values among all the chemical representations (0.510), surpassing AVN (0.489) and ECFP6 (0.460).All the other molecular descriptors exhibited lower performances, with APC being the lowest (0.388), with an *R*^2^ difference of 0.122 compared to AVN, as shown in Table 2 and Fig 2. Such differences were statistically significant, except when comparing RDF with AVN (p = 0.09) (2nd place) (see Table 8 for details).

Our findings are consistent with those of Sabando et al. [19], who compared molecular representations based on word embeddings with traditional approaches, such as MACCS and ECFP fingerprints, in both regression and classification tasks. Their study found no significant performance improvements when embedding methods were applied to QSAR modeling. Similarly, in our analysis, we observed an average ranking of 8.5 for the M2V embedding model, while RDF and AVN models achieved rankings of 2.06 and 3.25, respectively. Similarly, Orosz et al. [69] reported comparable predictive performance between MACCS and ECFP4 for pharmacodynamics and toxicity predictions using an XGB model. In our analysis, ECFP4 yielded an average *R*^2^ of 0.452, while MACCS produced a value of 0.404. However, the difference between these models was not statistically significant (p=0.06 ) according to the t-test (see Table 8). Lee et al. [70] further supported this notion by reporting similar average accuracies between MACCS and ECFP4 for biodegradation prediction. Xie et al. [71] demonstrated that combining ECFP4 fingerprints with MACCS keys can enhance the accuracy of predicting the LogP parameter, highlighting the complementary nature of these representations. However, as noted by Zagidullin et al. [72], model performance should also be evaluated using more qualitative considerations, such as downstream tasks and model characteristics, to fully capture and enhance the capabilities of these approaches.

The superior performance of RDF descriptors compared to substructural fingerprints like ECFP and MACCS suggests that predicting cancer bioactivity requires capturing more than just local substructural fragments. While binary fingerprints encode the presence or absence of specific groups, RDF descriptors capture the probability distribution of atomic properties at varying radial distances, effectively encoding steric constraints and inter-atomic relationships in a continuous manner [73]. This aligns with the findings of highly specific protein-ligand interactions, where the spatial arrangement of atoms, mimicking the pharmacophoric geometry of the binding pocket is often more predictive than topological connectivity alone [74]. By retaining information about the global topology and continuous electronic distribution, RDF descriptors likely avoid the “information loss” inherent in the bit-collision hashing processes of standard circular fingerprints.

The underwhelming performance of continuous embeddings like M2V relative to explicit descriptors suggests that unsupervised pre-training on general chemical space does not always translate to specific bioactivity endpoints. Continuous vector representations tend to smooth the feature space, which can obscure “activity cliffs” instances where a minor structural modification leads to a disproportionate change in potency [75]. Discrete descriptors and fingerprints are often better equipped to flag these specific structural alerts. Furthermore, learned representations typically require substantially larger training sets to fine-tune the embeddings for specific downstream tasks, suggesting that our dataset sizes were insufficient to leverage the full semantic power of the M2V architecture, as highlighted by Winter et al. [76].

From a practical standpoint, the trade-off between computational cost and predictive accuracy is a critical consideration for virtual screening campaigns. While RDF descriptors provided statistical improvements, they are computationally more intensive to generate than bit-vector fingerprints like ECFP4. Riniker and Landrum [42] demonstrated that circular fingerprints are among the fastest to compute, making them ideal for initial high-throughput screening of ultra-large libraries. Therefore, while RDF coupled with ETT offers the highest precision for lead optimization, a streamlined pipeline using ECFP-based XGB models may be more efficient for the early-stage filtering of millions of compounds, providing a balance between speed and acceptable predictive power [77].

Here, we evaluated the impact of some commonly used machine learning algorithms to generate models for QSAR. The algorithms, ranked by their *R*^2^ values, are ETT, HGT,RFT, XGB, SVR, etc., being ETT the best performer, as shown in Table 3 and Fig 12. The values for ETT did not present statistically significant differences from HGT (2^nd^ place), RFT (3^rd^) (which can be observed at Fig 12). At the same time, MLP and ENT showed the worst performance, with an *R*^2^ of 0.204 and 0.381, respectively. The preference for the RFT model in QSAR is due to its high predictive level and low number of adjustable parameters. At the same time, the XGB model is also recommended for its high accuracy and speed, where, regardless of the representation language, the RFT model has an average *R*^2^ value similar to XGB (0.530 and 0.521, respectively). The most effective combination was observed when usingAVN with SVR, as well as when using ECFP6 and ECFP4 with ETT models in Fig 4.

**Table 3:** Comparison of *R*^2^ and RMSE Performance Across Different Models. Values represent the coefficient of determination (*R*^2^) and RMSE. The highest-performing model (highest *R*^2^ and lowest RMSE) is indicated in bold.

Model	*R* ^2^	RMSE
BRG	0.4424	0.8027
ENT	0.3807	0.8538
ETT	**0.5408**	**0.7275**
HGT	0.5301	0.7408
*K*NN	0.4153	0.8348
LSS	0.3942	0.8458
MLP	0.2044	0.9668
PLS	0.3935	0.8489
RDG	0.4236	0.8282
RFT	0.5305	0.7346
SVR	0.4973	0.7534
XGB	0.5212	0.7447

The role of the ML model on the bioactivity prediction is highlighted by the works of Du et al. [78] and Wiriyarattanakul et al. [79]. In the former, different machine learning methods were evaluated for predicting the antioxidant activity of tripeptides, with *k*NN models outperforming XGB, RFT, and SVR in terms of accuracy (obtaining 0.996, 0.987, 0.945, and 0.926, respectively). Similarly, the latter conducted comparative studies for the QSAR prediction of anti-inflammatory activity, revealing a descending accuracy trend of SVM, GBR, and RFT, with accuracies of 0.907, 0.806, and 0.724, respectively. Similar results were found in inhibitor classification studies, where RFT demonstrated superior performance with an acurracy of 0.91 compared to PLS, which had an acurracy of 0.69 [80]. Additionally, for antibacterial compound classification, random forest and kNN achieved the highest accuracy (0.97) [81]. Lane et al. evaluated over 5,000 datasets using various algorithms for classification tasks, reporting SVC (0.796) and RFT (0.795) as the methods with better ROC-AUC, outperforming deep learning methods and kNN [82].

Our findings reinforce a growing consensus in the cheminformatics community: for tabular QSAR datasets of moderate size (N less than 100k), tree-based ensemble methods often outperform standard deep neural networks. The underperformance of the MLP model in this study (Average *R*^2^ = 0.202)) can be attributed to the lack of inductive bias suitable for tabular chemical data and the tendency of neural networks to overfit on smaller datasets without extensive pre-training [83]. Conversely, ensemble methods like ExtraTrees and XGBoost effectively handle high-dimensional feature spaces and reduce variance through bagging and randomization. As noted by Wu et al. [41], While deep learning dominates big data applications, traditional algorithms retain a distinct advantage in robustness and efficiency when applied to the limited datasets typical of bioactivity prediction.

Expanding upon our results, to assess the impact of the dataset on the *R*^2^ metric of the models, records of 15 therapeutic targets with more than 2,000 entries for breast, prostate, and lung cancer were obtained from the ChEMBL platform (Table 1). Crucially, the testing sets were generated using Murcko scaffold splitting [60]. Unlike random splitting, this approach ensures that the test compounds possess distinct molecular frameworks from the training set, thereby rigorously evaluating the models’ ability to generalize to new chemical spaces. Subsequently, we trained different machine learning models, which we later evaluated using various metrics. mTOR showed the highest *R*^2^ value (0.592), while AR had the worst performance (0.227), as seen in Table 4 and Fig 18.

**Table 4:** Comparison of $R^{2}\;$ and RMSE Performance Across Different Datasets. Values represent the coefficient of determination (*R*^2^) and RMSE. The highest-performing dataset (highest *R*^2^ and lowest RMSE) is indicated in bold.

Dataset	*R* ^2^	RMSE
HER2	0.5861	0.7898
HDAC6	0.4247	0.7756
AR	0.2270	0.7845
IGFR1	0.4233	**0.6699**
EGFR1	0.4241	0.8529
AURKB	0.2568	0.9552
FGFR3	0.0223	0.7347
VEGFR2	0.3466	0.8704
mTOR	**0.5920**	0.7661
PARP	0.5305	0.7490
PI3K	0.5273	0.7755
FGFR1	0.5288	0.7703
PI3K	0.5273	0.7755
AKT	0.5261	0.8493
CHK1	0.5822	0.8966
AURKA	0.5029	0.8975

The Pearson correlation coefficient between the dataset number and the average *R*^2^ was −0.03, which means no strong correlation was found between the dataset size and the *R*^2^ value. Significant effects of the dataset size on the performance metrics have been reported in classification tasks, as small datasets are not capable of capturing the population features, leading to overfitting, bias, poor generalization capabilities, and, in some cases, inaccurate predictions [84]. Further dataset analysis could enhance predictive performance, as the quality and content of the database can significantly influence the quality and validity of the QSAR model [85].

Finally, the variability in model performance across different cancer targets, ranging from an *R*^2^ of 0.0.592 for mTOR to 0.227 for AR, underscores the necessity of defining a rigorous Applicability Domain (AD) before deploying these models prospectively. As per OECD principles, a model’s predictive reliability is strictly limited to the chemical space defined by the training data [86]. The high disagreement observed in the AR and FGFR3 datasets suggests the presence of structural outliers or diverse binding modes that the models failed to generalize. Future implementation of these QSAR pipelines must incorporate AD assessment techniques, such as distance-to-model metrics or probability density estimation, to filter out unreliable predictions for compounds that are structurally dissimilar to the training set [87].

While this study provides valuable insights into the relationship between molecular representations and machine learning models in predicting bioactivity, several limitations may have influenced the findings. First, the datasets used, while relevant, are limited in size and chemical diversity. With only 15 cancer-related therapeutic targets, the data may only partially capture the broader chemical space, which could limit the generalizability of the models. A more diverse set of biological targets, combined with larger datasets, would be essential to improve the robustness of the results. Additionally, although RDF demonstrated strong performance, other molecular representations lagged. This may stem from their inability to capture molecular details as comprehensively as RDF, AVN and, ECFP6. Exploring other modern representations, such as 3D descriptors or graph neural network-based representations, could provide a broader evaluation of their impact on model performance.

Furthermore, the study focused primarily on traditional machine learning models, such as Random Forest and Support Vector Regressor, which may not fully exploit the complexity of molecular data compared to more advanced technique s like deep learning models. Future research could benefit from incorporating these newer models, such as graph neural networks or convolutional neural networks, which can capture more nuanced molecular interactions.

We acknowledge the importance of experimental validation in ensuring the reliability of bioactivity predictions. As noted, the current study relies on computational predictions based on available datasets such as ChEMBL, which, while informative, lack experimental confirmation. The absence of comparison with recognized inhibitors is indeed a limitation of the study. To address this concern, future work will include experimental validation, such as molecular docking studies or in vitro testing, to help validate the bioactivity predictions and explore potential mechanisms of action.

## Conclusion

After examining the impact of chemical representations in QSAR models, we conclude that RDF exhibited the best performance, followed by AVN and ECFP6. APC showed the least favorable performance, recommending their use as a supplement in combination with other representations. The performance of each representation may be influenced by other factors such as the choice of machine learning model, the dataset used, and the nature of the task. While RDF appears robust, it is essential to consider alternative representations and combinations, such as using descriptors as supplements to enhance model performance.

Regarding machine learning models, the ETT algorithm demonstrated the highest effectiveness, followed by HGT and RFT, with no statistically significant differences found between ETT and HGT/SVR, indicating that these algorithms might be more reliable for QSAR modeling. The dataset used also affected QSAR model performance, likely related to aspects such as quality and validity. A strong correlation was observed with AVN/ECFP4/ECFP6 chemical representations on ETT/HGT/SVR ($R^{2}\geq 0.7\;$). The acceptance of the alternative hypothesis in many cases suggests that QSAR model conditions (like representation language, preprocessing, and dataset) significantly impact performance, highlighting their importance in research.

Future work in QSAR modeling should focus on exploring novel chemical representations, such as 3D molecular descriptors, and advanced deep learning techniques like graph neural networks (GNNs) or convolutional neural networks (CNNs), which have the potential to capture intricate molecular interactions and spatial properties more effectively. Additionally, integrating multi-omics data (transcriptomics and proteomics) could significantly improve bioactivity predictions’ accuracy and contextual relevance. Ensemble modeling approaches could enhance model robustness and generalization, such as stacking different machine learning models and transfer learning techniques, which leverage pre-trained models on related tasks. Tackling issues like data imbalance and bias, incorporating explainable AI techniques to improve model interpretability, and exploring emerging areas such as quantum descriptors and quantum machine learning are promising directions for advancing QSAR research.
